# The genetics of cardiac failure: Role of a G protein-coupled receptor polymorphism in therapeutic response in an Indian population

**Published:** 2021-07-30

**Authors:** Sudha Ramalingam, Shanmugasundaram Radhakrishnan, Tamilarasu Kaliappan, Rajendiran Gopalan, Meenu Subrahmanian, Ramalingam Sankaran

**Affiliations:** ^1^PSG Center for Molecular Medicine and Therapeutics, PSG Institute of Medical Sciences and Research, Coimbatore, Tamil Nadu, India; ^2^Department of Cardiology, PSG Hospitals, Coimbatore, Tamil Nadu, India

**Keywords:** β-blocker, genotype, GRK5, heart failure, India, polymorphism

## Abstract

**Background and Aim:**

The incidence of heart failure (HF) is rising to epidemic proportions in developing countries like India. A lack of adequate Indian studies underscores the importance of pursuing research into HF in an Indian population. G protein-coupled receptor kinase 5 (*GRK5)* Gln41>Leu (rs2230345) polymorphism was reported as a genetic modifier associated with survival in HF patients. A prospective study was conducted to investigate the association of *GRK5* Gln41>Leu polymorphism with response to β-blocker therapy in Indian HF patients.

**Methods:**

HF patients (*n*=584) were recruited for the study. The patients were genotyped by tetra-primer based allele specific polymerase chain reaction and confirmed with Sanger sequencing. The HF patients were evaluated for *GRK5* gene expression and followed up for ~3 years. Drug dosages, cardiac output and hospitalization-free survival were evaluated as study outcomes. HF subgroups (i.e. systolic or diastolic dysfunction, biventricular dysfunction and pulmonary artery hypertension) were also analyzed in association with hospital-free survival.

**Results:**

HF patients showed genotype frequencies of AT (15%) and TT (1%). AT/TT genotype carriers showed downregulated *GRK5* gene expression and significant reduction in carvedilol drug dosage (*p=*0.0001). Moreover, AT/TT genotype carriers on β-blockers showed improved ejection fraction from 27% to 36% (*p*=0.0007) and increased hospitalization-free survival in comparison to other HF patients. HF patients with AA genotype showed an increased rate of hospital admission in comparison with patients with the AT/TT genotype. HF subgroups with the AT/TT genotype showed an increased hospitalization-free survival versus subgroups with the AA genotype.

**Conclusions:**

*GRK5* Gln41>Leu polymorphism in response to β-blocker therapy improved cardiac function in HF patients.

**Relevance for Patients:**

This study presents a comprehensive clinicofunctional pharmacogenetic characterization of *GRK5* Gln41>Leu polymorphism in a cohort of Indian HF patients. *GRK5* Gln41>Leu polymorphism can confer improved cardiac function and reduce hospitalization, thus improving the quality of life in HF patients.

## 1. Introduction

Heart failure (HF) is a major, multifactorial global health disease affecting approx. 6.5 million adults/year in the United States [1] and 491,600 – 1.8 million individuals/year in India [2]. The common etiologies of HF are coronary artery disease, hypertension, valvular heart disease, arrhythmia, dilated cardiomyopathy, infection, and inflammation [3]. Other risk factors include hypertension, ischemic heart disease, obesity, Type II diabetes mellitus, and rheumatic heart disease [2]. Growing evidence show that mortality rates as a result of HF are higher in Indian patients than in western countries with death records of 0.1-0.16 million individuals/year in India [4].

The use of beta (β)-adrenergic receptor (β-AR) antagonists continues to be the standard treatment for HF [5] and has effectively reduced mortality and morbidity rates over the past decade. β-AR, a G protein-coupled receptor expressed by cardiomyocytes has been documented as important for effective cardiac function [6]. Many studies have shown that elevated activation of β-AR results in abnormal β-AR signaling which leads to the pathogenesis of HF [7,8]. Therefore, much of the therapeutic drugs like β-blockers that target the β-AR, antagonize catecholamine-stimulated β-AR signaling [6] and have resulted in increased survival of HF patients.

As a regulator of β-AR signaling, G protein-coupled receptor kinases (GRKs) act by phosphorylating the activated β-AR, leading to receptor desensitization and diminished β-AR signaling [9]. Overexpression of GRK5 and GRK2 contribute to dysregulation of β-AR by excess desensitization and is also considered a hallmark of HF pathology [10]. Therefore, genetic polymorphisms in β-AR regulator genes may influence HF outcome. One such functional polymorphism being *GRK5* Gln41>Leu (rs2230345, c.122A>T, p.Q41L) in the *GRK5* gene. This nonsynonymous single nucleotide polymorphism (SNP) in the noncatalytic regulatory domain is prevalent in African Americans and confers a gain-of-desensitization function akin to the effects of β-blockade in cells. This polymorphism, however, did not alter the risk of HF but facilitated β-blocker response and reduced death [11,12].

We conducted a prospective study in a cohort of Indian HF patients to investigate the association of *GRK5*Gln41>Leu polymorphism in relation to β-blocker therapeutic response. The hypothesis behind this study was that as GRKs impose β-AR blockade by desensitization, the presence of genetic GRK variants might alter the outcome in HF patients. Therefore, the main aim of this study was to identify *GRK5* variants and to study their response to β-blocker treatment which might allow curtailing β-AR blocker usage in these selected patients to avoid potential harmful effects.

## 2. Materials and Methods

### 2.1. Study population

The study was conducted in multispecialty, tertiary care hospital. This prospective study recruited HF patients (*n*=584) with systolic or diastolic dysfunction, dilated cardiomyopathy or biventricular dysfunction (BVD) that attended the in- and outpatient cardiology clinic in our hospital. The study was initiated after obtaining the institutional ethics committee approval and getting informed consent from the patients. The HF patients considered for this study were diagnosed following the Framingham criteria [13]. The Framingham criteria for HF diagnosis should include either two major criteria or one major and two minor criteria. The major criteria included acute pulmonary edema, cardiomegaly, hepatojugular reflex, neck vein distention, paroxysmal nocturnal dyspnea or orthopnea, pulmonary rales, and third heart sound (S3 gallop rhythm). The minor criteria included ankle edema, dyspnea on exertion, hepatomegaly, nocturnal cough, pleural effusion, and tachycardia (heart rate >120 beats/min). Healthy controls (*n*=60) without any comorbidity were recruited for gene expression studies.

Stage-appropriate medications given to HF patients included diuretics, angiotensin-converting enzyme (ACE) inhibitors or β-blockers or angiotensin receptor blockers (ARBs) alone or in combination. If needed, mineralocorticoid receptor antagonists and hydralazine were also included. β-blockers were determined by the treating physicians (60% carvedilol, 17% metoprolol, 12.5% nebivolol, and 10.5% bisoprolol) and defined as continuous therapy for at least 6 months. β-blocker dosages were monitored during the follow-up period. Initial dosage refers to the dosage administered at the start of the β-blocker therapy after the diagnosis of HF. The final dosage refers to the dosage followed at the last follow-up time. The number of hospitalizations was monitored for each patient and the follow-up data were collected for 3-7 years. The mean follow-up duration was 3.7 years and 3.5 years for the β-blocker group and non-β-blocker group.

### 2.2. Clinical and echocardiography assessment

A complete resting two-dimensional and M-mode echocardiography color flow and spectral Doppler as well as annular tissue Doppler imaging data were obtained from the subjects using a Philips IE33™ ultrasound system (Philips Medical Systems, Eindhoven, The Netherlands). Standard two-dimensional parasternal short-axis and apical 2- and 4-chamber views were recorded. Left ventricular (LV) ejection fraction (EF) was calculated using the Teichholz formula. Mitral Doppler signals were recorded in apical 4-chamber view and early diastolic mitral inflow peak velocity (E), late diastolic mitral inflow peak velocity (A), and E/A ratio were calculated. Thus, confirmation of HF in patients was determined on clinical and echocardiography assessment. Echocardiographic parameters were measured according to current recommendations [14].

### 2.3. Genotyping

Blood samples from the patients were collected by venipuncture in EDTA coated vials and immediately aliquoted and frozen at −80°C. Genomic DNA was isolated using EZ-10 spin column blood genomic DNA purification kit (Bio Basic Inc., Markham, Ontario, Canada). *GRK5* (c.122A>T, p.Q41L) polymorphism was screened and confirmed through in-house developed tetra-primer based allele specific polymerase chain reaction (PCR) and confirmed with restriction fragment length polymorphism (RFLP) method and Sanger sequencing. PCR reaction mixture containing 100 ng of DNA, Taq polymerase mix (Ampliqon, Odense, Denmark), primers for tetra primer PCR (35nM GRK5F primer: 5’- GAAGGCTGTGGTCTAGTCTAGGGGAACAG - 3’and GRK5R primer: 5’- TTGATTAGCAAAAAGGTCAGCCTGGAGC - 3’, 45nM SNPTF primer: 5’ - TGGCTAATGTGAGGGAAC - 3’and SNPTR primer: 5’ -TGTGTGAAGACCTCCGA - 3’) or primers for RFLP method (50 nM BSRF primer: 5’- CTACAGCCCGTCCCTCTGT - 3’and BSRR primer: 5’ - GCAAAAAGGTCAGCCTGGAGC - 3’) were subjected to thermal cycling (Eppendorf, Hamburg, Germany). For RFLP, PCR product was digested using 1.5 units of *Bsr*I (New England Biolabs^®^ Inc., Ipswich, Massachusetts, USA) as per manufacture instructions. The genotype was identified for tetra-primer PCR (AA: 580 bp and 225 bp, AT:580 bp, 380 bp and 225 bp, and TT:580 bp and 380 bp) and RFLP method (AA:202 bp and 179 bp, AT:381 bp, 202 bp and 179 bp, and undigested TT:381 bp) with these banding patterns after resolving on the gel.

### 2.4. Quantitative PCR (qPCR)

RNA was isolated from the HF patients and unaffected controls using the Aurum™ total RNA mini kit (Bio-Rad, Hercules, California, USA). The RNA was qualitatively verified on denaturing formaldehyde 1.2% agarose gel electrophoresis. Using 1 μg of RNA, respective cDNAs was synthesized using iScript™first strand cDNA synthesis kit (Bio-Rad, Hercules, California, USA). qPCR was carried out to compare *GRK5* gene expression in HF cases and healthy controls using relative quantification with β-actin as reference gene. PCR mixture of 20μL contained 2X SYBR green (Sso Advanced universal SYBR^®^ Green supermix, Bio-Rad, Hercules, California, USA), 50 nM of forward and the reverse primer of the respective gene, cDNA template corresponding to 1 μg RNA and nuclease free water. The primers used were GRK5 FP (5’ - ACCTGAGGGGAGAACCATTC - 3’), GRK5 RP (5’ -TGGACTCCCCTTTCCTCTTT - 3’), ACTIN FP (5’ - TCCCTGGAGAAGAGCTACG - 3’), and ACTIN RP (5’ - GTAGTTTCGTGGATGCCACA - 3’). Reaction included an initial denaturation of 95°C for 3 min followed by 45 cycles of 95°C for 30 s, 60°C for 30 s, 72°C for 45 s, and a melt curve analysis (65 – 95°C) was performed at the end of 45 cycles. Normalized fold expression was calculated using 2^–ΔΔCt^ method.

### 2.5. Variables

In this study, the independent variables were age, sex, and socio-demographic data. Covariates were (EF%), comorbidities, use of β-blockers, and use of other medications. The covariates adjusted for hospital-free survival analysis were age, sex, EF%, and comorbidities.

### 2.6. Statistical analysis

Student *t*-tests (paired and unpaired) and Chi-square tests were used to assess significant differences in variables between the groups, drug dosages, genotype classes, gene expression, and EF%. Continuous variables are expressed as mean ± standard deviation (SD). Fischer’s exact test was carried out to compare cardiac output between groups. Hardy-Weinburg equilibrium was assessed to compare predictions with that of the obtained results. Kaplan–Meier curves were also constructed to plot the percentage of survival of patients. Comparisons of overall survival were performed using a log-rank (Mantel-Cox) test. All the statistical analyses were carried out using GraphPad Prism software version 5.0 Windows (GraphPad Software, San Diego, California, USA).

## 3. Results

### 3.1. Clinical and demographic characteristics of the HF patient cohort

The HF patient cohort (*n*=584) was considered for the study after clinical and echocardiographic assessment. Table 1 shows the clinical and demographic characteristics of the HF patient cohort. The HF patients were categorized into β-blocker group and non-β-blocker group. There was no statistically significant difference in variables such as age, sex, body mass index, and hemodynamics between the two groups. BVD (24%; *p*=0.002) and LV diastolic dysfunction (32.8%; *p*=0.002) were observed as statistically significant HF characteristics in the β-blocker group. Dilated cardiomyopathy (54%; *p*=0.0002) was observed as statistically significant HF etiology in the β-blocker group versus the non-β-blocker group whereas rheumatic heart disease (8%; *p*=0.0001) and coronary artery disease (44%; *p*=0.0001) were significantly higher in the non-β-blocker group. Type II diabetes mellitus (60%; *p*=0.0001) and its associated disorders were significantly frequent in the non-β-blocker group whereas systemic hypertension (42%; *p*=0.002) and dyslipidemia (25%; *p*=0.002) were higher in the β-blocker group. Approximately 52% of the patients were on β-blocker therapy. Among the medications administered, 38% of the HF patients received ACE inhibitors, 20% ARBs, 70% diuretics, 35% digitalis glycosides, 22% statins, and 19% anticoagulants.

**Table 1:** Clinical and demographic characteristics of the HF patient cohort

Variables	HF patient cohort (*n*=584)	

	β-blocker group (*n*=304)	Non-β-blocker group (*n*=280)
Mean Age±SD (years) (range)	60±10 (24 – 89)	59±11 (25 – 90)
Males (%)	73	73
Mean body mass index (kg/m^2^) (range)	22 (19.5 – 24.5)	23.5 (21.1 – 26.5)
Mean follow up time (years) (range)	3.7 (3 – 7)	3.5 (3 – 7)
Hemodynamics		
Heart rate (beats/min)	78	80
Mean systolic blood pressure (mmHg)	128 (81 – 171)	123 (80 – 170)
Mean diastolic blood pressure (mmHg)	84 (50 – 139)	72 (55 – 130)
HF Etiology, n (%)		
Coronary artery disease	89 (26)	131 (47)*
Dilated cardiomyopathy	164 (54)	109 (36)*
Rheumatic heart disease	0	24 (8)*
HF Characteristics, n (%)		
LV systolic dysfunction	106 (35)	112 (40)
LV diastolic dysfunction	100 (32.8)	50 (18)*
Biventricular dysfunction	74 (24)	40 (14)*
Pulmonary artery hypertension	52 (17)	50 (18)
Comorbidities, n (%)		
Type II diabetes mellitus and its associated disorders	106 (35)	168 (60)*
Systemic hypertension	128 (42)	84 (30)*
Dyslipidemia	76 (25)	42 (15)*
Hypothyroidism	49 (16)	56 (20)
Cerebrovascular events	18 (6)	12 (4)
Renal insufficiency	9 (3)	14 (5)
Anemia	6 (2)	6 (2)
β-blocker use, n (%)		
Carvedilol	183 (60)	-
Metoprolol	52 (17)	-
Nebivolol	38 (12.5)	-
Bisoprolol	32 (10.5)	-
Other medications (%)		
ACE inhibitors (angiotensin-converting enzyme)	103 (34)	118 (42)*
ARB (angiotensin receptor blocker)	0	56 (20)*
Diuretics	170 (56)	241 (86)*
Digitalis glycosides	91 (30)	112 (40)*
Statins	31 (10)	98 (35)*
Anticoagulants	31 (10)	56 (20)*

* *p*≤0.05 versus β-blocker group.

### 3.2. GRK5 Gln41>Leu polymorphism in HF patient cohort

**Figure 1:**
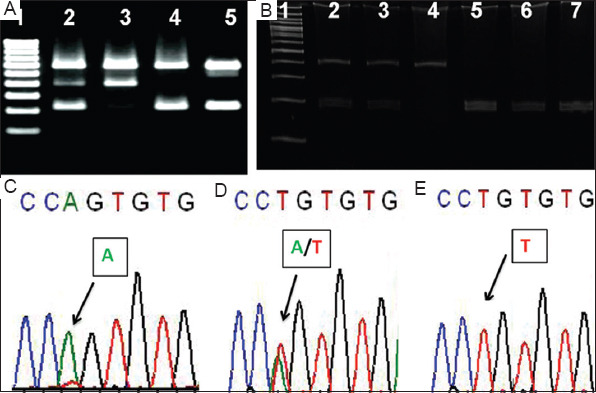
Genotyping of GRK5 Gln41>Leu polymorphism. Tetra-primer based allele specific polymerase chain reaction (A) results on showing banding pattern for Gln/Leu heterozygous AT genotype (lane 2: 580 bp, 380 bp and 225 bp), Leu/Leu homozygous TT genotype (lane 3: 580 bp and 380 bp), Gln/Gln homozygous AA genotype (lanes 4, 5: 580 bp and 225 bp). RFLP (B) results showing banding pattern for Gln/Leu heterozygous AT genotype (lanes 2, 3: 381 bp, 202 bp and 179 bp), Leu/Leu homozygous TT genotype (lane 4: 381 bp) and Gln/Gln homozygous AA genotype (lanes 5, 6, 7: 202 bp and 179 bp). Lanes 1a, 1 b: 100 bp DNA ladder (Biobasic, Canada). Sanger sequencing results of Gln/Gln homozygous AA genotype C), Gln/Leu heterozygous AT genotype (D) and Leu/Leu homozygous TT genotype (E).

*GRK5* Gln41>Leu polymorphism was screened by in-house developed tetra-primer based allele specific PCR and reconfirmed with RFLP and the Sanger sequencing method (Figure 1). Genotype distribution was in agreement with the Hardy-Weinberg equilibrium (*p*=0.595). The genotype (84% for AA, 15% for AT, and 1% for TT) and allele frequencies (0.916 for A allele and 0.084 for T allele) of *GRK5* Gln41>Leu polymorphism in the HF patient cohort is presented in Table 2. On comparing the genotype frequencies, AT genotype was more prevalent in the β-blocker group (19%) compared to the non-β-blocker group (10%) (*p*=0.011). The unaffected healthy controls were also screened for this polymorphism. The genotype and the allele frequencies of the healthy controls (Supplementary Table 1) did not differ significantly from the HF cases (*p*=0.94 for genotype frequency, *p*=0.82 for allele frequency) ensuring a lack of association of this polymorphism with development of HF.

**Table 2:** *GRK5* genotype and allele frequencies in the HF patient cohort

Genotype	HF patients (*n*=584), *n* (%)	HF patients (β-blockers) (*n*=304), *n* (%)	HF patients (non-β-blockers) (*n*=280), *n* (%)	*p*-value
AA	493 (84)	244(80)	249(89)	0.011
AT	85 (15)	57 (19)	28 (10)	
TT	6 (1)	3 (1)	3 (1)	
Allele				0.07
A	535 (91.6)	273 (90)	263 (94)	
T	49 (8.4)	31 (10)	17 (6)	

Results were expressed as *n* (%) for genotype and allele distribution. χ2 test was significant for genotype frequency and not significant for allele frequency between β-blocker group and the non-β-blocker group.

As the frequency of homozygous TT genotype was low, we intended to divide the HF patients into two group, those having AA genotype alone (AA genotype group) and those with AT and TT genotype (AT/TT genotype group), to study the influence of *GRK5* Gln41Leu (rs17098707) polymorphism on response to β-blocker therapy in the HF patients.

### 3.3. Downregulated GRK5 expression among the GRK5 Leu41 variants (AT/TT genotype)

**Figure 2:**
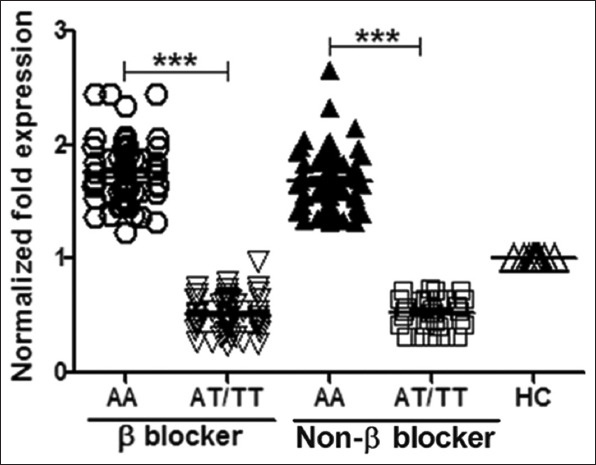
*GRK5* expression among different groups of HF patients. β-blocker AT/TT group versus β-blocker AA group (*p*<0.0001), non-β-blocker AT/TT group versus non-β-blocker AA group (*p*<0.0001) along with healthy controls (HC) were analyzed for *GRK5* normalized gene expression. Student’s *t*-test was used to compare the groups.

The *GRK5* gene expression was evaluated among different groups of HF patients. The gene expression among The AT/TT genotype carriers showed a downregulated *GRK5* expression than AA genotype carriers among β-blockers and non-β-blockers. For gene expression analysis (Figure 2), healthy controls (*n*=60) were included for comparison. The β-blocker AT/TT group (*n*=60) showed a 0.5135±0.1593-fold decrease in gene expression and the β-blocker AA group (*n*=60) showed a slightly but significant upregulated pattern of 1.752±0.0.2775 gene expression (*p*<0.0001). On comparing the non-β-blockers, there was a significant difference in the gene expression (*p*<0.0001) between the AT/TT group (*n*=31, 0.5507±0.1322) and the AA group (*n*=60, 1.681±0.2688).

### 3.4. Improvement in the EF of the GRK5 Leu41 variants (AT/TT genotype)

**Figure 3:**
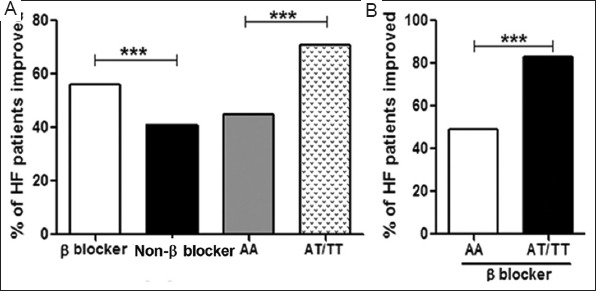
Percentage improvement in cardiac output among the cohort of HF patients. The percentage of HF patients improved among β-blocker and non-β-blocker groups (*p*=0.0004) and different genotype groups of *GRK5* (*p*=0.0001) (A). The percentage of HF patients improved among β-blocker group with different genotypes (*p*=0.0001) (B). Fischer’s exact test was carried out for analysis.

The EF% is the measure of LV systolic function and cardiac output. Among the β-blocker group (29±4.8%) and non-β-blocker group (41.5±2.3%), the mean EF% remained significantly varied (*p*=0.0286). The EF% of the patients before and after pharmacological treatment was recorded and improvement in their cardiac output was compared among the groups (Figure 3). The increment in the EF% after treatment was considered as an improvement in cardiac output. About 56% of HF patients on β-blockers and 41% on non-β-blockers showed improvement in their EF% (*p*=0.0004; Figure 3A). As expected, 71% of the *GRK5* Leu41 variants (AT/TT genotype) showed remarkably higher cardiac output (*p*=0.0001; Figure 3A) versus those with *GRK5*Gln41 (AA genotype, 45%). Since the non-β-blockers had HF preserved EF, only the β-blocker group having HF reduced EF was considered for further analysis. When the HF patients with different genotypes in the β-blocker group were compared it was evident that those with the AT/TT genotype (83%) improved significantly (*p*=0.0001; Figure 3B) compared to those with the AA genotype (49%).

**Figure 4:**
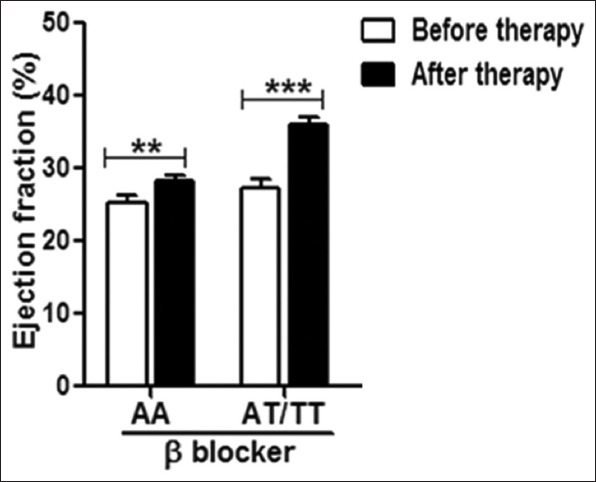
Improvement in ejection fraction in HF patient cohort. The improvement in EF% in HF patients with different genotypes of β-blocker group was measured. AT/TT group (*p*=0.0091) improved significantly in comparison with the AA group (*p*=0.0091). Paired *t*-test was carried out for computing *p*-values.

The improvement in cardiac output was compared among the HF patients in β-blocker group with different genotypes (Figure 4). EF% has improved from 25.2±1.041% to 28.2±0.7638% among the AA group (*p=*0.0091) and 27.4±1.021% to 36±1% in the AT/TT group (*p=*0.0007).

### 3.5. GRK5 Leu41 variants (AT/TT genotype) show decrement in β-blocker drug dosage

**Figure 5:**
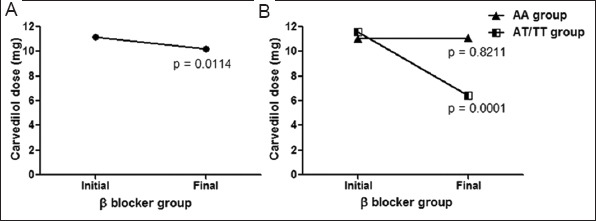
GRK5 Gln41>Leu polymorphism influences β-blocker (carvedilol) drug dosage. Carvedilol drug doses were reduced in the overall β-blocker group (*p*=0.0114) (A). The drug doses remained unchanged in the AA genotype group (*p*=0.8211), whereas in the AT/TT genotype group there was a significant reduction in the drug dosage (*p*=0.0001) (B). Paired *t*-test was used to compare the drug doses between groups.

Among the β-blocker group (*n*=304), 60% of the HF patients were treated with carvedilol, 17% with metoprolol, 12.5% and 10% with nebivolol and Bisoprolol, respectively. The initial and the final drug dosages over the follow-up period were noted and analyzed. Initial dosages administered at the start of the β-blocker therapy and final dosages followed at the last follow-up were noted. There was a statistically significant reduction in carvedilol drug dosage in the overall β-blocker group when the initial (11.2±7.7 mg) and final drug dosages (10.2±7.1 mg) were compared (*p=*0.0114; Figure 5A). Furthermore, the drug dosages remained unchanged in the AA genotype group (*p=*0.8211; Figure 5B) when the initial (11.06±7.3 mg) and final (11.1±7.4 mg) drug dosages were compared. Interestingly, there was a statistically significant reduction in the drug dosage in the AT/TT genotype group (*p=*0.0001; Figure 5B) when the initial (11.6±9.2 mg) and the final (6.4±4 mg) drug doses were compared. Thus, the *GRK5* Leu41 variants in the homozygous and heterozygous forms were associated with a lower dose of β-blockers.

### 3.5. Reduced event of hospitalization among the GRK5 Leu41 variants (AT/TT genotype)

**Figure 6:**
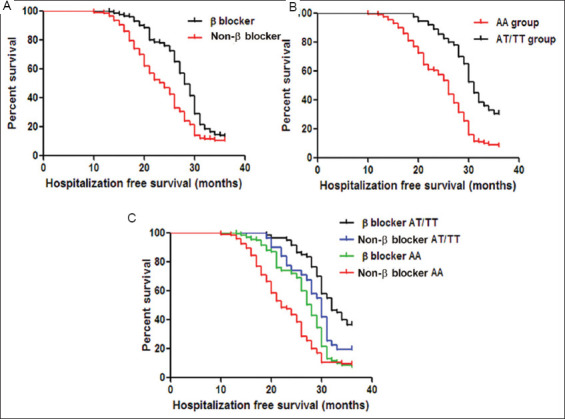
Influence of β-blocker therapy and *GRK5* genotype on hospital-free survival of HF patients. HF patients were stratified by β-blockers usage (β-blockers (*n*=304); non-β-blockers (*n*=280); *p*<0.0001) (A). HF patients were stratified by *GRK5* Leu41 carrier status (AT/TT genotype) (GRK5 AA group (*n*=493); *GRK5* AT/TT genotype (*n*=91); *p*<0.0001) (B). β-blocker treated subjects and non-β-blocker subjects stratified by *GRK5* Leu41 carrier status (AT/TT genotype) (β-blocker AA group (*n*=244); β-blocker AT/TT group(*n*=60); non-β-blocker AA group(*n*=249); non-β-blocker AT/TT group (*n*=31) (C). Log-rank test (Mantel-Cox) test was utilized for comparisons of survival curves.

The HF patients were followed-up for ~3 years (40 months) after the initiation of pharmacological treatment. The influence of β-blocker therapy and *GRK5* genotype on hospitalization-free survival of HF patients was studied through Kaplan–Meier survival curves (time-to-event analysis). When the overall HF patients administered with β-blockers and non-β-blockers were compared for hospital-free survival, the patients on β-blockers showed higher survival than the non-β-blockers (hazard ratio [HR]: 1.167, 95% confidence interval [CI]: 0.325 – 2.008, log-rank *p*<0.0001; Figure 6A). The same was found when the overall HF patients with AA and AT/TT genotypes were compared; the presence of AT/TT genotype reduced the risk for hospitalization (HR: 0.8387, 95% CI: 0.07042 – 1.607, log-rank *p*<0.0001; Figure 6B). On further analysis of the HF subgroups, β-blocker AT/TT group versus β-blocker AA group, it was found that the presence of AA genotype increased the risk for hospitalization (HR: 1.143, 95% CI: 0.4337 – 1.852, log-rank *p*<0.0001; Figure 6C) even under the influence of β-blocker therapy. When non-β-blocker AT/TT group versus non-β-blocker AA group was analyzed, AA genotype increased the risk for hospitalization (HR: 1.364, 95% CI: 0.7020 – 2.025, log-rank *p*=0.0003; Figure 6C). In the β-blocker AT/TT group versus non-β-blocker AT/TT group analysis, the use of non-β-blockers increased the risk for hospitalization (HR: 1.067, 95% CI: 0.463 – 1.670, log-rank *p=*0.0231; Figure 6C). As expected, when β-blocker AA group versus non-β-blocker AA group was analyzed, the presence of AA genotype and use of non-β-blocker therapy increased the risk of hospitalization (HR: 1.273, 95% CI: 0.441 – 2.104, log-rank *p*<0.0001; Figure 6C).

**Figure 7:**
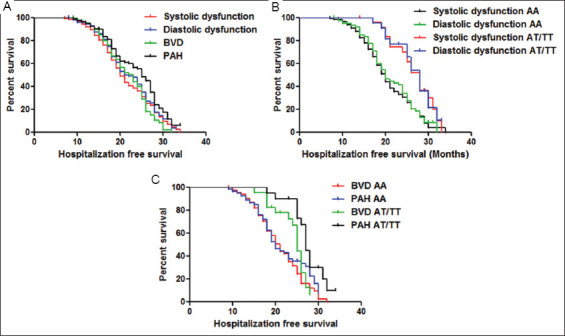
Influence of *GRK5* genotype on hospital-free survival of HF patient subgroups. HF patients were stratified by HF characteristics systolic dysfunction (*n*=218); diastolic dysfunction (*n*=150); BVD (*n*=114); PAH (*n*=102); *p*=0.02) (A). HF patients with systolic and diastolic dysfunction were stratified by *GRK5* Leu41 carrier status (AT/TT genotype) (systolic dysfunction AA group (*n*=193); diastolic dysfunction AA group (*n*=128); systolic dysfunction AT/TT group (*n*=25); diastolic dysfunction AT/TT group (*n*=22); *p*=0.0002) (B). HF patients with BVD and PAH were stratified by *GRK5* Leu41 carrier status (AT/TT genotype) (BVD AA group (*n*=90); PAH AA group (*n*=82); BVD AT/TT group (*n*=24); PAH AT/TT group (*n*=20); *p*=0.0051) (C). Log-rank test (Mantel-Cox) test was utilized for comparisons of survival curves.

The overall HF patients were subdivided based on HF characteristics such as systolic dysfunction (*n*=218), diastolic dysfunction (*n*=150), BVD (*n*=114), and pulmonary artery hypertension (PAH) (*n*=102). The groups were compared for hospitalization-free survival. The patients with PAH showed higher survival and patients with systolic dysfunction showed the lower survival than the other groups (log-rank *p*=0.02; Figure 7A). When the subgroups with systolic dysfunction and diastolic dysfunction were compared with *GRK5* polymorphism genotypes (AA and AT/TT), it was evident that the HF patients with AA genotype under both the subgroups indicated the lower hospitalization-free survival than the HF patients with AT/TT genotype under both subgroups (log-rank *p*=0.0002; Figure 7B). When the subgroups with BVD and PAH were compared with *GRK5* polymorphism genotypes (AA and AT/TT), it was evident that the HF patients with AA genotype under both the subgroups indicated lower hospitalization-free survival than those with the AT/TT genotype under both subgroups (log-rank *p*=0.0051; Figure 7C).

**Figure 8:**
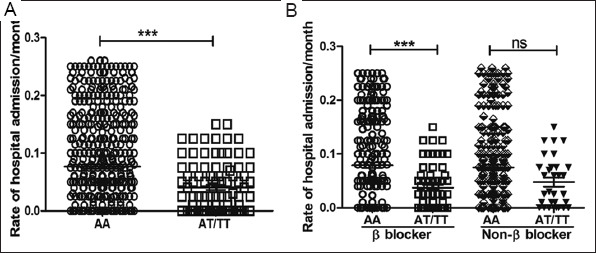
Comparison of frequency of hospital admission between the genotype groups. The AA group of HF patients (*n*=493) had more hospital admissions than the AT/TT HF group (*n*=91; *p*<0.0001) (A). Among the β-locker group, HF patients with the AA genotype had more frequent hospital admission than the AT/TT variants (*p*=0.0002) (B). A similar trend was observed among the non-β-blocker group but the results were no statistically insignificant (ns). β-blocker AA group (*n*=244); β-blocker AT/TT group (*n*=60); non-β-blocker AA group(*n*=249); and non-β-blocker AT/TT group (*n*=31). Student’s *t*-test was used to compare the gr.

To compare the frequency of hospital admissions between the genotype groups within a month, the rate of hospital admission/month was compared (Figure 8). From the results, it was evident that the HF patients in the AA group (0.0769±0.003; *n*=493) were significantly more frequently (*p*<0.0001) admitted in hospital than the AT/TT group (0.0409±0.004; *n*=91; Figure 8A). Among the β-blocker group, HF patients with the AA genotype (0.0790±0.005; *n*=244) were significantly more frequently admitted in the hospital than the AT/TT variants (0.0374±0.005; *n*=60; *p*=0.0002). Among the non-β-blocker group, HF patients with the AA genotype (0.0749±0.005; *n*=249) had more hospital admissions compared to the AT/TT variants (0.0477±0.008; *n*=31) although not statistically significant (*p*=0.0674; Figure 8B).

## 4. Discussion

Genetic polymorphisms impact HF outcomes. One such polymorphism is *GRK5*Gln41>Leu (A>T), which was proven to have a role in promoting β-AR desensitization [11]. Our results show that HF patients with *GRK5* Leu41 variant in its homozygous and heterozygous forms show decreased events in hospitalization and specifically respond to β-blockers and require reduced β-blocker drug dosage in comparison to *GRK5* Gln41 carriers. Moreover, *GRK5* Leu41 variants in combination with β-blocker therapy showed improved cardiac output.

The *GRK5* genotype frequencies in this study (AT/TT15% and TT1%) were comparatively lower than that reported in African Americans (AT/TT39% and TT6%) [15]. The T allele frequency in our study (0.084) was lower than African Americans (0.23) [15] but, higher than that found in the Chinese population (0.009) [16] and Caucasians (0.017) [17]. As GRK5 is highly expressed in myocardium [18], and given its established role in cardiac failure pathology [10], this prompted our group to examine and evaluate *GRK5* expression in a cohort of Indian HF patients. GRK5 is reportedly overexpressed in HF conditions and could attenuate cardiac burden in response to adrenergic surge [19,20]. In view of this, a slight upregulation observed in *GRK5* expression in a subpopulation of HF patients could be expected. However, gene expression was downregulated in the AT/TT subset of HF patients in our study. A possible reason could be that the role of overexpressed GRK5 in β-AR desensitization is compensated by the allelic variation, bringing about an augmented desensitization, thus attempting to regulate *GRK5* expression for better cardiac performance. Interestingly though inhibiting GRK5 expression restores the cardiac muscle damage incurred during HF [21]. These observations support the fact that *GRK5* Leu41 variant confers protection from congestive HF [22].

Human and rodent data have proven that *GRK5*Leu41 protected against death or cardiac transplantation [11]. Coinciding with these data, our study reaffirms that the *GRK5*Leu41 allele improved quality of life, promoted hospitalization-free survival of South Indian HF patients. Independently, both the administration of β-blocker therapy and the presence of Leu41 allele yielded a positive outcome on hospitalization-free survival. The Leu41 allele carriers among the different HF subgroups showed increased hospitalization-free survival in comparison to Gln41 carriers. The previous reports support the fact that Indians are highly sensitive to β-blockers [23] and also speculated that polymorphic variations in β-AR gene in Indians [24] might be the reason for higher sensitivity [25]. Our data indicate that the prevalence of *GRK5* gene polymorphisms in Indian HF patients and its role in cardiac failure might be the reason for responders to β-blocker treatment. In response to β-blocker treatment, *GRK5*Leu41 allele carriers (AT/TT genotype) showed better hospital-free survival than *GRK5* Gln41 allele carriers (AA genotype) and β-blocker naïve *GRK5*Leu41 and Gln41 allele carriers. This observation was also reported in a study where HF patients with the Gln41Leu genotype had reduced hospitalization rates (18.6%) [26]. These results also justify the continued use of β-blockers as standard therapy for cardiac failure and their benefits over other pharmacological interventions [27-29]. Interestingly, it was found that although Leu41 carriers among African Americans survived better than Gln41 carriers, they exhibited a reduced response to β-blockers [11,17] such as atenolol [30]. Another study among African race demonstrated that Leu41 impacted negatively on survival of HF patients, but the effect seemed to be stabilized upon β-blocker usage. In other words, Gln41 carriers survived better in the presence and absence of β-blockers [15].

The Leu41 allele seemed to act as a genetic β-blockade [11], promoting reduced dependency on β-blockers for improvement in HF patients. Although β-blocker has been validated as a lifesaving drug in HF, in view of its adverse effects such as HF deterioration, hypotension, and impaired left atrial function [31,32], minimizing its usage is recommended. In our study, the presence of Leu41 allele has helped in curtailing the dosage of carvedilol during the course of treatment and reduced the frequency of hospital admission compared to the Gln41 allele. Carvedilol, a nonselective β-blocker with better efficacy than other β-blockers [10,33] was used in 60% of the HF patients in this study. The usage of β-blockers has substantially improved the LV systolic function by increasing the EF in 56% of HF patients in this study. Literature also supports the usage of β-blockers in recovering from LV systolic dysfunction [34]. The improvement in EF was pronounced in *GRK5* Leu41 variants compared to Gln41 carriers and this observation coincided with another study reporting *GRK5* polymorphism having a positive effect on systolic HF after β-blocker therapy [16]. Taken together, these data present convincing evidence that the Leu41 allele and β-blocker therapy synergistically work to stabilize cardiac function in Indian HF subjects.

### Ethical Approval

Human samples to carry out the study were obtained following informed consent of patients according to the Declaration of Helsinki and its later amendments or comparable ethical standards. Research involving humans was authorized by the Institutional Ethics committee from PSG Institute of Medical Sciences and Research, Coimbatore, Tamil Nadu, India.

## Appendix

**Supplementary Table 1:** *GRK5* genotype and allele frequencies in the HF patients and healthy controls

Genotype	HF patients (*n*=584), *n* (%)	Healthy controls (*n*=400), *n* (%)	*p* value
AA	492 (84)	340 (85)	0.94
AT	86 (15)	56 (14)	
TT	6 (1)	4 (1)	
Allele		
A	535 (91.6)	368 (92)	0.82
T	49 (8.4)	32 (8)	

Results were expressed as *n* (%) for genotype and allele distribution. χ^2^ test was non significant for genotype and allele frequencies between HF patient cohort and healthy controls group.
